# Organ-specific cholesterol metabolic aberration fuels liver metastasis of colorectal cancer

**DOI:** 10.7150/thno.55609

**Published:** 2021-04-27

**Authors:** Kai-Li Zhang, Wen-Wei Zhu, Sheng-Hao Wang, Chao Gao, Jun-Jie Pan, Zun-Guo Du, Lu Lu, Hu-Liang Jia, Qiong-Zhu Dong, Jin-Hong Chen, Ming Lu, Lun-Xiu Qin

**Affiliations:** 1 Department of General Surgery, Huashan Hospital & Cancer Metastasis Institute & Institutes of Biomedical Sciences, Fudan University, Shanghai, 200040, China.; 2 Department of Pathology, Huashan Hospital, Fudan University, Shanghai, 200040, China.

**Keywords:** colorectal cancer, liver metastasis, SREBP2, cholesterol biosynthesis, HGF

## Abstract

**Rationale:** Metastasis, the development of secondary malignant growth at a distance from a primary tumor, is the main cause of cancer-associated death. However, little is known about how metastatic cancer cells adapt to and colonize in the new organ environment. Here we sought to investigate the functional mechanism of cholesterol metabolic aberration in colorectal carcinoma (CRC) liver metastasis.

**Methods:** The expression of cholesterol metabolism-related genes in primary colorectal tumors (PT) and paired liver metastases (LM) were examined by RT-PCR. The role of SREBP2-dependent cholesterol biosynthesis pathway in cell growth and CRC liver metastasis were determined by SREBP2 silencing in CRC cell lines and experimental metastasis models including, intra-splenic injection models and liver orthotropic injection model. Growth factors treatment and co-culture experiment were performed to reveal the mechanism underlying the up-regulation of SREBP2 in CRC liver metastases. The *in vivo* efficacy of inhibition of cholesterol biosynthesis pathway by betulin or simvastatin were evaluated in experimental metastasis models.

**Results:** In the present study, we identify a colorectal cancer (CRC) liver metastasis-specific cholesterol metabolic pathway involving the activation of SREBP2-dependent cholesterol biosynthesis, which is required for the colonization and growth of metastatic CRC cells in the liver. Inhibiting this cholesterol biosynthesis pathway suppresses CRC liver metastasis. Mechanically, hepatocyte growth factor (HGF) from liver environment activates SREBP2-dependent cholesterol biosynthesis pathway by activating c-Met/PI3K/AKT/mTOR axis in CRC cells.

**Conclusion:** Our findings support the notion that CRC liver metastases show a specific cholesterol metabolic aberration. Targeting this cholesterol biosynthesis pathway could be a promising treatment for CRC liver metastasis.

## Introduction

Metastatic disease remains the leading cause of cancer related death 1. Metastasis is a complex multistep process involving dissemination and intravasation of primary tumor cells, survival of disseminated tumor cells (DTCs) in circulation, colonization of DTCs in distant metastatic organ, and the subsequent proliferation of these colonies into clinically detectable metastatic lesions, which are regulated by a complex network of intra- and inter-cellular signal transduction cascades 2, 3. Organ-tropism is an important feature of cancer metastasis, many cancers exhibit metastatic preference to specific organs 4. Although “seed and soil” theory have been proposed for more than 100 years 5, the mechanism through which DTCs adapt to and colonize a new organ environment, the crucial steps of the metastasis process, is still largely undetermined.

Altered metabolism is one of the hallmarks of cancer cells. Cancer cells are known to undergo many metabolic alterations to sustain faster proliferation 6. In addition to its role in primary tumors, metabolic reprogramming is also essential to the metastatic process 7. Recent studies have showed that DTCs undergo metabolic adaptation in metastatic organ microenvironment, which is increasingly recognized as a pivotal step in cancer metastasis 8, 9. The metabolic alternations of DTCs also exhibit metastatic organ specificity in many solid tumors, supporting the survival and colonization of DTCs in different metastatic organs 10-13.

Colorectal cancer (CRC) is one of the most common malignant tumors worldwide 14. Approximately 50% of CRC patients will develop liver metastasis during tumor progression 15. However, only a small part of patients with metastatic CRC are eligible for surgical intervention, other effective treatment modalities are limited, and these therapies for metastatic CRC do not specifically target liver metastases, making liver metastasis a leading cause of death for CRC patients 16, 17. Emerging evidences have shown that metastatic CRC cells undergo adaptive alterations in the liver, which is essential for the colonization and growth of the metastatic CRC cells 18, 19. However, the metabolic alterations of metastatic CRC cells are still largely undetermined.

Metastatic CRC cells is reported to gain a liver-specific gene transcription program in the liver 20. Liver is the central organ for cholesterol homeostasis, about a half of de novo cholesterol synthesis of the body occurs in the liver 21, 22. Cholesterol is an essential component of mammalian cell membrane, as well as the precursor of bile acids and steroid hormones 23, 24. The de novo biosynthesis of cholesterol from acetyl-CoA plays an important role in tumor growth and progression 25. Cholesterol biosynthesis is controlled by Sterol regulatory element-binding proteins 2 (SREBP2), a key transcription factor in cholesterol biosynthesis, and its downstream genes including 3-hydroxy-3-methylglutaryl-CoA reductase (*HMGCR*), 3-hydroxy-3-methylglutaryl-CoA synthase 1 (*HMGCS*), and squalene synthase (*SS*) 26. Whether DTCs of CRC undergo cholesterol metabolic alternations to adapt to liver microenvironment is unknown.

In this study, we identified a CRC liver metastasis-specific cholesterol metabolic pathway involving the activation of SREBP2-dependent cholesterol biosynthesis, which is required for the colonization growth of CRC in the liver. Then, we showed the effect of inhibiting this specific cholesterol metabolic pathway on growth of CRC liver metastases, and further revealed the mechanism underlying the activation of this specific cholesterol metabolic pathway in liver metastases of CRC.

## Methods

### Materials

Tissue samples including colorectal tumors, adjacent non-tumorous colorectal tissues, liver metastases, and adjacent non-tumorous liver tissues were collected from 17 patients with colorectal cancers and liver metastases (Table S1). Seven paired samples of colorectal primary tumors and brain metastases were obtained from CRC patients with only brain metastases. In addition, 5 pairs of colorectal tumors and lymph node metastases were from CRC patients with only lymph node metastasis. Clinical samples were collected from patients after obtaining informed consent in accordance with a protocol approved by the Ethics Committee of Huashan hospital, Fudan University (Shanghai, China).

### Cell lines

CRC cell lines (SW620, HT29, LoVo, DLD-1 and HCT116), human embryonic kidney 293T cells, human normal liver cell line L02, human hepatic stellate cell line LX2 were obtained from Cell Bank of Chinese Academy of Sciences (Shanghai, China). LoVo were maintained in F12 (Gibco) supplemented with 10% (v/v) FBS at 37 °C in 5% CO_2_. Other cell lines were maintained in DMEM (Gibco) supplemented with 10% (v/v) FBS at 37 °C in 5% CO_2_. For cell proliferation and cholesterol determination experiments, DMEM supplemented with 5% (v/v) lipoprotein-deficient serum (LPDS) was used.

### Antibodies

The primary antibodies include anti-β-actin (Santa Cruz), anti-GAPDH (ABclonal), anti-SREBP2 (Abcam, for western blotting), anti-SREBP2 (Santa Cruz sc-271616 for immunohistochemistry), anti-mTOR (Cell Signaling Technology), anti-phospho-mTOR-ser2481 (Cell Signaling Technology), anti-phospho-mTOR-ser2448 (Cell Signaling Technology), anti-MET (Cell Signaling Technology), anti-phospho-MET (Cell Signaling Technology), anti-AKT (Cell Signaling Technology), anti-Phospho-Akt (Ser473) (Cell Signaling Technology), anti-Phospho-Akt (Thr308) (Cell Signaling Technology), anti-p70 S6 Kinase (Cell Signaling Technology), anti-Phospho-p70 S6 Kinase (Cell Signaling Technology). HGF neutralizing antibody was purchased from R&D Systems, Inc.

### Inhibitors and growth factors

Betulin, Simvastatin and Rapamycin were purchased from Selleck Chemicals. Betulin or simvastatin was used to treat cells at indicated concentrations for 24 h. Rapamycin was added with/without HGF to treated cells at indicated concentration for 24 h. DMSO was used as the vehicle control. Cholesterol was purchased from Sigma, dissolved in ethanol. In cholesterol rescue experiment, cholesterol was used to treat cells at 0, 500 or 1 mg/mL for 24 h. The human recombinant human growth factors, including hepatocyte growth factor (HGF), epidermal growth factor (EGF), vascular endothelial growth factor (VEGF) and insulin-like growth factor (IGF) were purchased from PeproTech, and were dissolved in DMEM and were used to treat cells at indicated concentrations for 24 h.

### Immunohistochemistry

Tissue samples were prepared and preserved through paraffin embedding and were mounted onto 3-aminopropyltrioxysilane-coated slides, dewaxed and blocked in hydrogen peroxide/methanol solution. Antigen retrieval was performed by pressure-cooking the sample in 0.08% citrate buffer for 20 min. Before it was used on the tissue section, the antibody was tittered against normal control tissues to determine the dilutions that rendered optimal sensitivity and specificity. Staining results were visualized by sequential incubations with the components of the Envision-plus detection system (EnVision + /HRP/Mo, Dako, Glostrup, DK) and 3,3'-diaminobenidine. Negative controls were treated in the same way without adding the primary antibody. Immunohistochemistry staining was assessed by two independent pathologists with no prior knowledge of the patient characteristics.

Discrepancies were resolved by consensus. The staining extent score was on a scale of 1 to 4, corresponding to the percentage of immunoreactive tumor cells and the staining intensity. IHC H-score was computed as the sum of 4 × (% 4) + 3 × (% 3) + 2 × (% 2) + 1 × (% 1).

### RNA extraction and quantitative real-time PCR (qRT-PCR)

Total RNA was extracted from cultured cells and tissue samples using the TRIzol reagent (#15596-018, Life Technologies) and reverse-transcribed to cDNA using the Super-Script III First-Strand Synthesis System (#18080-051, Life Technologies). Gene expression was analyzed in triplicate using FastStart Universal SYBR Green Master with ROX (#04913850001, Roche Applied Science) on the Applied Biosystems 7900HT Fast Real-Time PCR System. Relative expression of each gene was calculated according to the 2^-ΔΔCt^ method. β-actin was used as an internal control for normalization. Primers used for qRT-PCR were listed in Supplementary Table 2.

### Western blot

Cells were lysed in the RIPA buffer with protease inhibitor cocktail (Selleck) and phosphatase inhibitor cocktail (Roche), and used to perform Western blot analysis. Briefly, cell lysates were denatured by boiling at 95 °C for 5 min and resolved by sodium dodecyl sulfate polyacrylamide gel electrophoresis (SDS-PAGE). Proteins were transferred to NC membrane and blocked with 5% non-fat milk in TBS buffer with 0.1% Tween-20 (TBST) at room temperature for 1 h. The membranes were then sliced according to the molecular weights and incubated with primary antibodies at 4 °C overnight, washed with TBST, and incubated with horseradish peroxidase (HRP)-conjugated secondary antibodies at 4 °C overnight. Signals were detected with Image Acquisition using ImageQuant LAS 4000.

### Lipoprotein-deficient serum preparation and cholesterol determination

Lipoprotein-deficient serum (LPDS, density < 1215 mg/mL) was prepared from FBS by ultracentrifugation 27. For determining the total cholesterol levels in cells or tissue samples, they were first harvested in RIPA buffer, sonicated, and were extracted with chloroform/methanol (2:1; v/v). The chloroform phase was separated, dried, and dissolved in ethanol. Total cholesterol levels were then determined using Amplex Red Cholesterol Assay Kit (Thermo Fisher, A12216) according to the manufacturer's instructions. The blood total cholesterol levels were determined with an assay kit from FUJIFILM Wako Diagnostics according to the manufacturer's instructions.

### Lentivirus-mediated knockdown of SREBP2 expression

Short hairpin RNAs (shRNAs) targeting SREBP2 and scramble sequences were cloned into pLKO.1-puro vector (Addgene). The sequences of PCR primers and shRNA sequences were provided in Table S3. For lentivirus generation, 1 × 10^7^ 293T cells were seeded in 10-cm dish in DMEM supplemented with 10% FBS the day before transfection. Cells were transfected by changing to 10 mL of DMEM containing 16 µL of Lipofectamine 2000 (Thermo Fisher Scientific), 4 µg of pLKO.1 shscrambled or pLKO.1 shSREBP2, combined with 3 µg psPAX2 and 1 µg pMD2.G. 6 h later, media was changed by DMEM with 10% FBS. The supernatant was recovered, filtered with 0.45 μm filters and used to infect CRC cell lines. The stable transfected cell lines were obtained by puromycin selection for 1 week.

### Cell proliferation and colony formation assays

HT29, SW620 and their corresponding stable transfected cell lines were first seeded in DMEM supplemented with 10% FBS overnight for cell adherence, and the medium was then changed to medium A (containing betulin or not) for 72 h.

For cell proliferation assay, cells (1000 per well) were seeded on a 96-well plate and CCK8 assay was carried out daily over a 5-day course to evaluate cell proliferation. At the end of each culture period, the viable cells were measured according to the manufacturer's protocol. Cells were incubated in 10% CCK-8 (Dojindo Molecular Technologies, Gaithersburg, MD, USA) diluted in normal culture medium for an additional 1h. The absorbance at a wavelength of 450 nm was used to estimate the viable cells in each well.

For colony formation assays, 500 cells were plated in triplicate on a six-well plate and cultured for two weeks refreshing medium every two days. At the end of the incubation, the cells were fixed with 1% paraformaldehyde for 30 min and stained with 0.1% (w/v) crystal violet for 30 min. The numbers of cell colonies were counted using Image-Pro Plus 5.0 software (Media Cybernetics, Bethesda, MD, USA).

### Co-culture experiment

Co-culture experiment was performed using 6-well transwell apparatus with 0.4 µm pore size (Costar) according to the manufacturer's instructions. CRC cells (1 × 10^5^) were added into the upper chamber, and L02, LX2 or 293T cells (1 × 10^6^) were added into the lower chamber. These cells were co-cultured for another 48 hours and then lysed for mRNA and protein samples.

### Mouse models

HT29 shNT cells or HT29 shSREBP2 cells (1 × 10^6^) were subcutaneously implanted into both flanks of nude mice (5 mice per group) to establish subcutaneous tumor models. Tumor volumes were assessed on indicated days by a caliper and calculated using the formula: volume = (Length × Width^2^) / 2.

When the volume of subcutaneous HT29 xenograft tumors reached to 0.5 cm^3^, the tumors were removed and dissected into 1-mm^3^ sections. These tumor sections were incubated into cecum of nude mice to establish cecum orthotopic xenograft models.

To establish intra-splenic injection models, HT29 cells (1 × 10^6^/200 μL PBS) were injected into spleen and observed for 6 weeks.

Different HT29 stable cell lines (1 × 10^6^) were injected under the liver capsule (Glisson's capsule) of mice to establish liver orthotropic injection model. For drug treatment experiment, after 3 days, mice were randomly separated into four groups (five per group) were treated by intragastric administration with vehicle control, simvastatin (30 mg/kg/day), or betulin (15 or 30 mg/kg/day) daily. At the end of each procedure (about 6 weeks), mice were sacrificed, and tumors were dissected, weighed, taken photos, and used for different experiments.

All experiments were performed in 6 weeks old male nude mice (BALB/c nu/nu) obtained from Shanghai SLAC Laboratory Animal Co., Ltd (Shanghai, China) and maintained on a 12 h light dark cycle at 22 ºC. All mice were treated in strict accordance with animal care procedures and methods approved by the institutional review board of Department of Laboratory Animal Science, Fudan University, and conformed to the guidelines of National Institutes of Health on the ethical use of animals.

### Statistical analysis

Experimental data were analyzed as indicated in figure legends. Statistical significance was evaluated with GraphPad Prism 5.01 (GraphPad Software). P-values of < 0.05 were considered statistically significant.

## Results

### Cholesterol biosynthesis pathway is activated specifically in liver metastases of CRC

**Figure 1:**
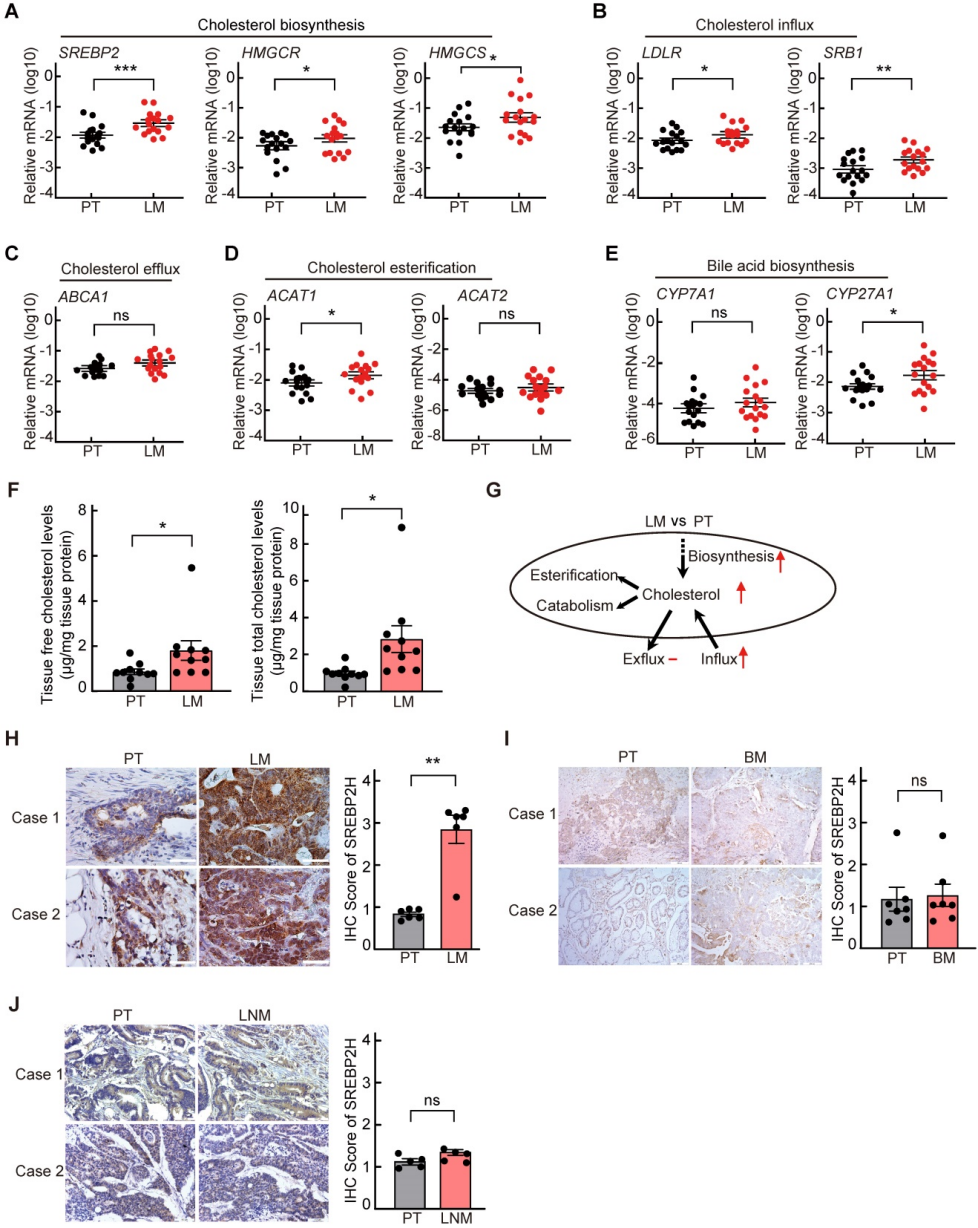
** Cholesterol biosynthesis metabolic pathway is specifically activated in liver metastasis of colorectal cancer. A-E,** Quantitative RT-PCR analysis of mRNA levels for cholesterol metabolism-related genes in 17 paired samples from patients diagnosed with primary colorectal cancer and liver metastasis. Primary tumors (PT) and paired liver metastases (LM). Data are shown as mean ± SEM after log transformation (n = 17). Each dot represents the mean of relative mRNA levels (log10) in triplicates for the indicated gene in each tissue sample. **F,** Free and total cholesterol contents in PT and LM. Data are shown as mean ± SEM (n = 10). **G,** A schematic diagram depicts the expression changes of key genes involved in cholesterol metabolism in LM versus PT. **H-J,** Representative immunohistochemistry (IHC) pictures of SREBP2 protein in paired CRC samples of PT and LM (n = 6) (left of H), PT and brain metastases (BM) (n = 7) (left of I), or PT and lymph node metastases (LNM) (n = 5) (left of J). The average IHC scores were shown in right of H-J, data were shown as mean ± SEM. Scale bar, 100 μm. Significance was determined by two-tailed ratio *t*-test (A-F and right of H-J). *P < 0.05, **P < 0.01, ***P < 0.001, ns, not significant.

To explore the cholesterol metabolic alterations in liver metastasis of CRC, the expressions of key genes involved in cholesterol metabolism were first analyzed in 17 paired CRC samples of primary tumors (PTs) and corresponding liver metastases (LMs) by quantitative reverse-transcription PCR (qRT-PCR). We found that the mRNA levels of key genes involved in cholesterol biosynthesis, including *SREBP2* and its downstream target gene *HMGCS* and *HMGCR*, in LM were significantly higher than those in the paired PT (Figure 1A). Among the other cholesterol metabolic genes examined, the mRNA levels of *LDLR* and *SRB1*, which are also the downstream target genes of *SREBP2* and are responsible for cholesterol influx^26^, were also significantly upregulated in LMs (Figure 1B-E). While the expressions of most genes involved in glucose metabolism and lipid metabolism were not significantly changed in LMs compared to their PTs (Figure S1). The up regulation of *SREBP2* in LMs were also observed in four published GEO datasets (Figure S2A). To further validate the activation of SREBP2-dependent cholesterol biosynthesis pathway, we examined the cholesterol levels in PTs and LMs, and found that both total and free cholesterol levels in LMs were significantly higher than those in PTs (Figure 1F). These results indicated that cholesterol biosynthesis pathway is activated in CRC liver metastases (Figure 1G).

Next, to determine whether the activation of cholesterol biosynthesis pathway is specifically in liver metastasis of CRC, we further detected the SREBP2 protein levels in PTs and their paired LMs, brain metastases (BMs) or lymph node metastases (LNMs) of CRC by immunohistochemistry (IHC). The protein levels of SREBP2 were much higher in LMs than those in PTs (Figure 1H), which is consistent with the mRNA levels detected by qRT-PCR above (Figure 1A). While SREBP2 protein levels were not significantly changed in BM (Figure 1I) or in LNM (Figure 1J), in comparison with their paired PT, suggesting that the activation of cholesterol biosynthesis pathway is specific for liver metastasis. Furthermore, the expression of SREBP2 did not show significant difference between CRC primary tumors and paired non-tumor tissues (Figure S2B-C), suggesting that the up regulation of SREBP2 does not occur in primary tumor stage.

**Figure 2:**
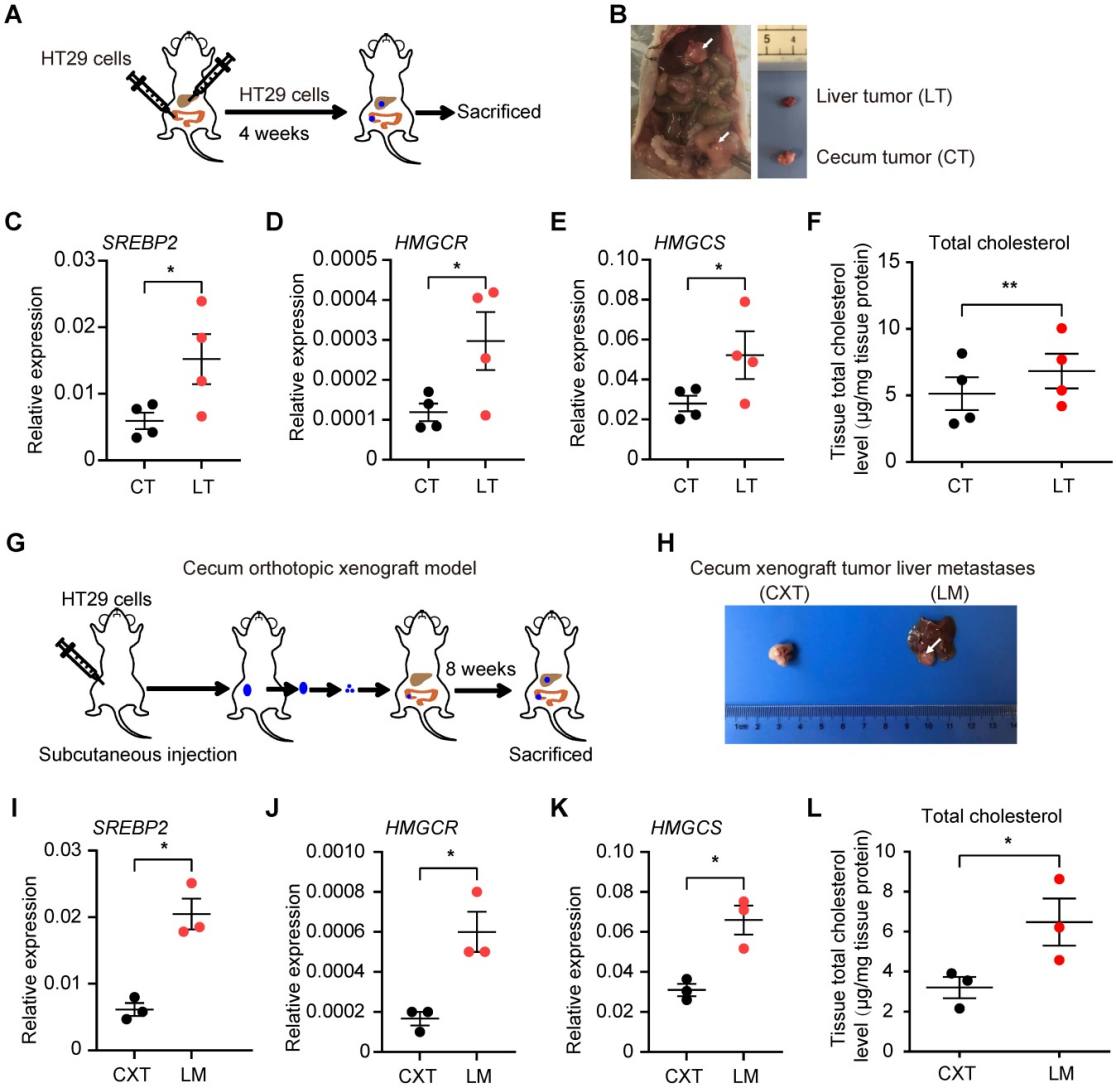
** Cholesterol biosynthesis pathway is induced in CRC liver metastasis *in vivo*. A,** Schematic of the experimental CRC liver metastasis model by liver orthotropic injection. **B,** Representative images of liver tumor (LT) and cecum tumor (CT) from CRC liver metastasis model by liver orthotropic injection. **C-E,** The expression of SREBP2, HMGCR and HMGCS genes in LT and CT determined by RT-qPCR analysis. Each dot represents the relative mRNA level in LT or CT from a mouse (n = 4). **F,** Cholesterol detection showing accumulation of total cholesterol in liver metastases compared with primary cecum tumors. Each dot represents the cholesterol level in LT or CT from a mouse (n = 4). **G,** Schematic of the experimental CRC liver metastasis model by orthotopic cecum injection. **H,** Representative images of cecum xenograft tumor (CXT) and liver metastases (LM) from CRC liver metastasis model by orthotopic cecum injection. **I-K,** The expression of SREBP2, HMGCR and HMGCS genes in CXT and LM determined by RT-qPCR analysis. Each dot represents the relative mRNA level in CXT or LM from a mouse (n = 3). **L,** Cholesterol detection showing accumulation of total cholesterol in liver metastases compared with primary cecum tumors. Each dot represents the cholesterol level in CXT or LM from a mouse (n = 3). Significance was determined by a two-tailed paired t-test (C-F, I-L). *P < 0.05, **P < 0.01.

To further validate these findings in CRC liver metastases, we implanted HT29 cells (a CRC cell line) into the mouse liver and cecum simultaneously. CRC tumors formed in liver and cecum, and we harvested the respective tumors four weeks after the injection (Figure 2A-B). qRT-PCR analysis confirmed increased SREBP2, HMGCR and HMGCS mRNA levels in CRC tumors from the liver than those from the cecum (Figure 2C-E). Total cholesterol level in CRC tumors from the liver was also higher than that from the cecum (Figure 2F). We then utilized cecum orthotopic xenograft model, and the elevated expression of SREBP2, HMGCR and HMGCS genes as well as increased total cholesterol level in liver metastases tissues could also be observed (Figure 2G-L). These results above indicate that SREBP2-dependent cholesterol biosynthesis pathway is activated specifically in CRC liver metastases.

### Cholesterol biosynthesis pathway is required for CRC cell growth

**Figure 3:**
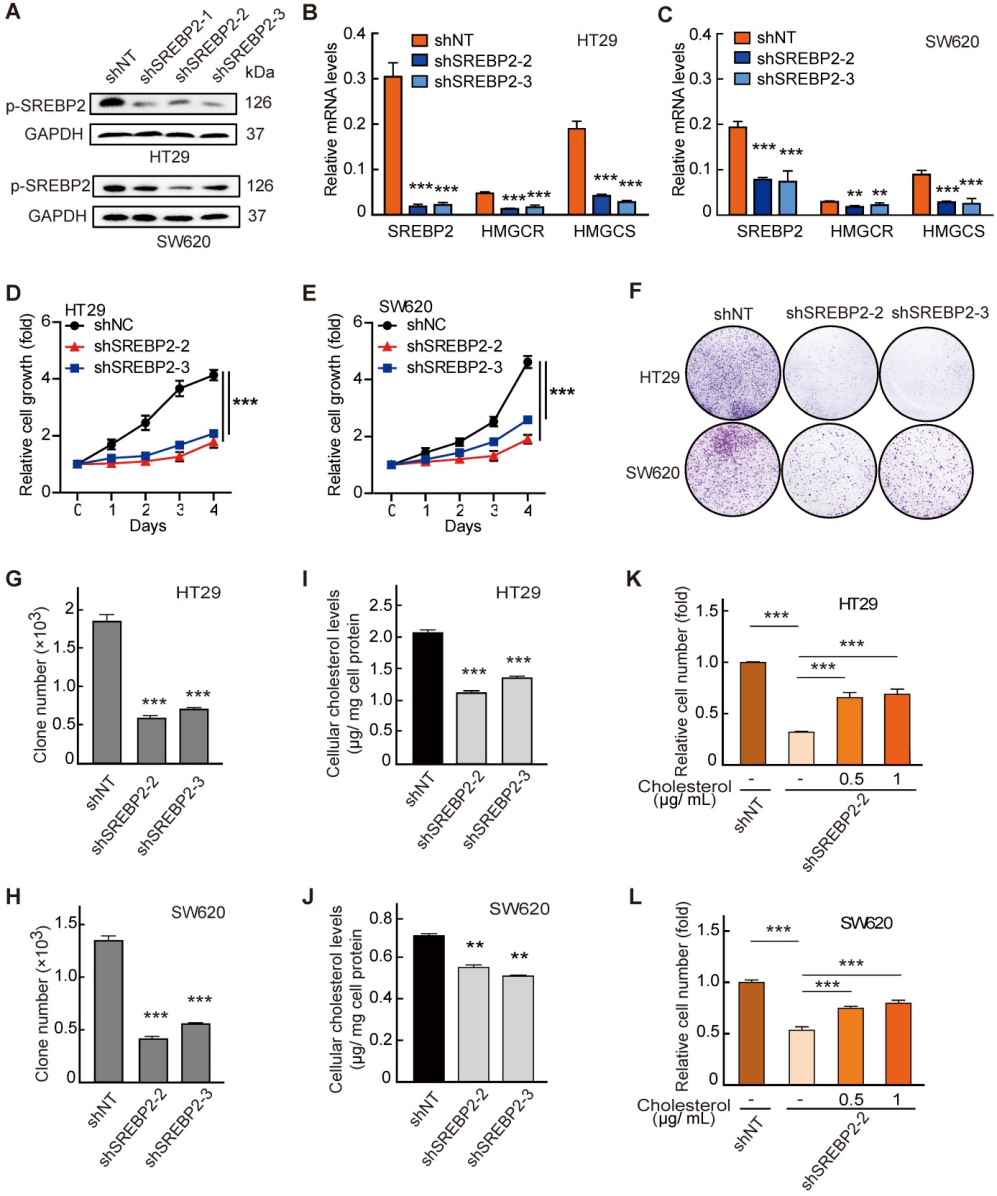
** Cholesterol biosynthesis pathway is required for CRC cell growth. A-C,** Confirmation of SREBP2 knockdown in HT29 cells and SW620 cells determined by western blotting (A) and quantitative RT-PCR (B and C). **D, E,** The effect of SREBP2 knockdown on cell proliferation in HT29 cells (D) and SW620 cells (E). The relative cell number was expressed as fold change to Day 0. **F-H,** The effect of SREBP2 knockdown on colony formation in HT29 cells and SW620 cells. Briefly, 500 cells were plated in triplicate on a six-well plate and cultured for two weeks. The representative photo is shown in (F). **I, J,** The effect of SREBP2 knockdown on total cholesterol levels in HT29 cells (I) and SW620 cells (J). Representative results from at least three independent experiments are shown. **K, L,** cholesterol supplementation reverses the effect of SREBP2 knockdown on cell proliferation in HT29 cells (K) and SW620 cells (L). SREBP2 knockdown HT29 cells were treated at 0, 0.5 or 1 µg/mL for 24 h. Data are shown as mean ± SD of triplicate or quadruple experiments (B-E and G-L). Significance was determined by two-way ANOVA. *P < 0.05, **P < 0.01, ***P < 0.001.

Next, to determine the functional role of SREBP2 in the* in vitro* proliferation of CRC cell lines, we generated three *SREBP2*-specific short hairpin (sh) RNAs to silence the SREBP2 expression (sh*SREBP2*). The sh*SREBP2*#2 and sh*SREBP2*#3 induced the most significant knockdown effects in HT29 and SW620 cells that highly express SREBP2 (Figure 3A and Figure S3), and were adopted for knocking down the SREBP2 expression in the following studies. SREBP2 knockdown was further validated by the decreased mRNA levels of its downstream target genes *HMGCR* and *HMGCS* (Figure 3B-C). Knockdown of SREBP2 led to a dramatic inhibition of *in vitro* cell proliferation of both HT29 (Figure 3D) and SW620 cells (Figure 3E). Significant decreases on colony formation could also be observed in SREBP2 knockdown HT29 and SW620 cells (Figure 3F-H). We then measured the cellular cholesterol levels and found that knockdown of SREBP2 resulted in obvious reductions on total cholesterol levels in both HT29 and SW620 cells (Figure 3I-J). We further verified that whether SREBP2 knockdown caused inhibition of cell growth was resulted from its effect on cellular cholesterol level by replenishing cholesterol in SREBP2 knockdown cells. The results showed that cholesterol replenishment hindered the effect of SREBP2 knockdown on suppressing cell growth of both HT29 and SW620 cells (Figure 3K-L). We further confirmed the role of SREBP2-dependent cholesterol biosynthesis in tumor growth *in vivo*. In subcutaneous xenograft models, silencing of SREBP2 significantly suppressed the tumor growth (Figure S4A-F). Furthermore, the expression of SREBP2 and the total cholesterol levels were examined in CRC xenograft tissues, and the results indicated that the knockdown of SREBP2 was maintained in HT29 and SW620 xenograft tumors (Figure S4G-J). These *in vitro* and *in vivo* results demonstrate that SREBP2-dependent cholesterol biosynthesis is required for CRC growth.

### Cholesterol biosynthesis pathway is required for liver colonization of metastatic CRC cells

**Figure 4:**
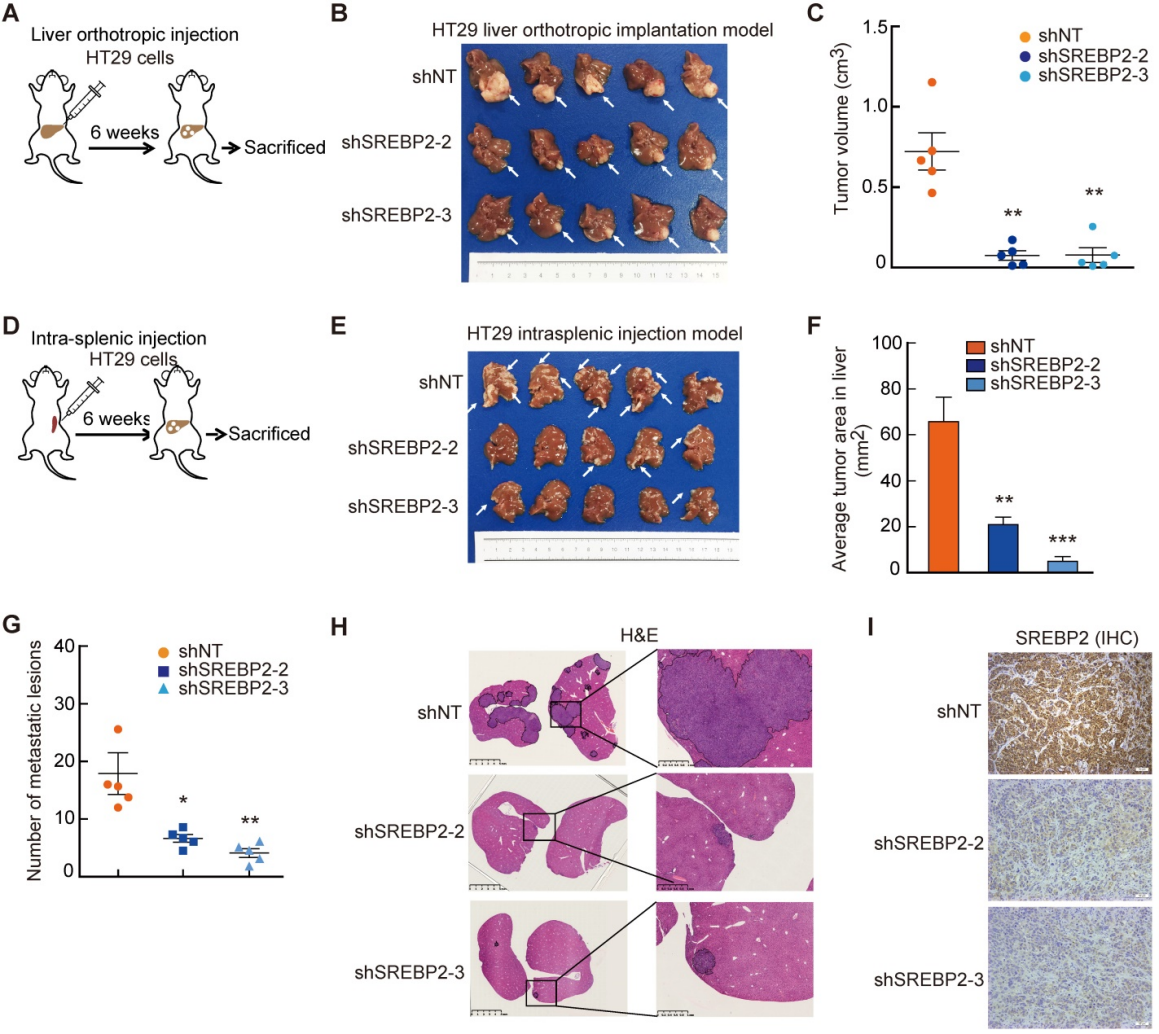
** Cholesterol biosynthesis pathway is required for CRC liver metastasis *in vivo.* A-C,** The effect of SREBP2 silencing on CRC liver metastasis in liver orthotropic injection model. Different HT29 stable cell lines were injected into liver of mice (A). At the end of the experiment, liver metastases were taken photos (B), and were dissected for assessing tumor volume (C). **D-I,** The effect of SREBP2 silencing on CRC liver metastasis in intrasplenic injection model. Schematic diagram of intrasplenic injection mice model was presented in (D) Different HT29 stable cell lines were intrasplenically injected into nude mice. At the end of the experiment, liver metastases were taken photos (E), and were dissected for assessing tumor area (F) and metastatic lesion number (G), or for H&E staining (H) and IHC staining (I) of SREBP2 protein. Scale bar, 50 μm. For (C), (F) and (G), Data are shown as mean ± SEM (n = 5). Significance was determined by one-way ANOVA. *P < 0.05, **P < 0.01, ***P < 0.001.

To investigate the role of SREBP2-dependent cholesterol biosynthesis pathway in CRC liver metastasis, we first established experimental CRC liver metastasis model by direct intrahepatic injection (Figure 4A). Notably, the liver xenograft tumors derived from HT29 shSREBP2 cells were much smaller than those from HT29 shNT cells (Figure 4B-C). Similar results could also be observed in xenograft tumors derived from SW620 cells (Figure S5). To further explore the role of SREBP2-dependent cholesterol biosynthesis in colonization of metastatic CRC cells in the liver, we utilized another experimental CRC liver metastasis model by intrasplenic injection of HT29 cells transfected with non-target shRNA control (shNT) or HT29 SREBP2 knockdown cells using BALB/c nude mice (Figure 4D). Mice were sacrificed after 6 weeks and the livers were removed for evaluating liver metastasis. Both the number and the size of liver metastases of the shSREBP2 group were significantly decreased compared with those of the shNT group (Figure 4E-H). Furthermore, IHC staining showed that knockdown of SREBP2 was maintained in liver metastases (Figure 4I). These results demonstrate that knockdown of SREBP2 could significantly inhibit the *in vivo* colonization and growth of CRC liver metastases.

### HGF induces cholesterol biosynthesis pathway in CRC liver metastases by activating c-Met/mTOR pathway

**Figure 5:**
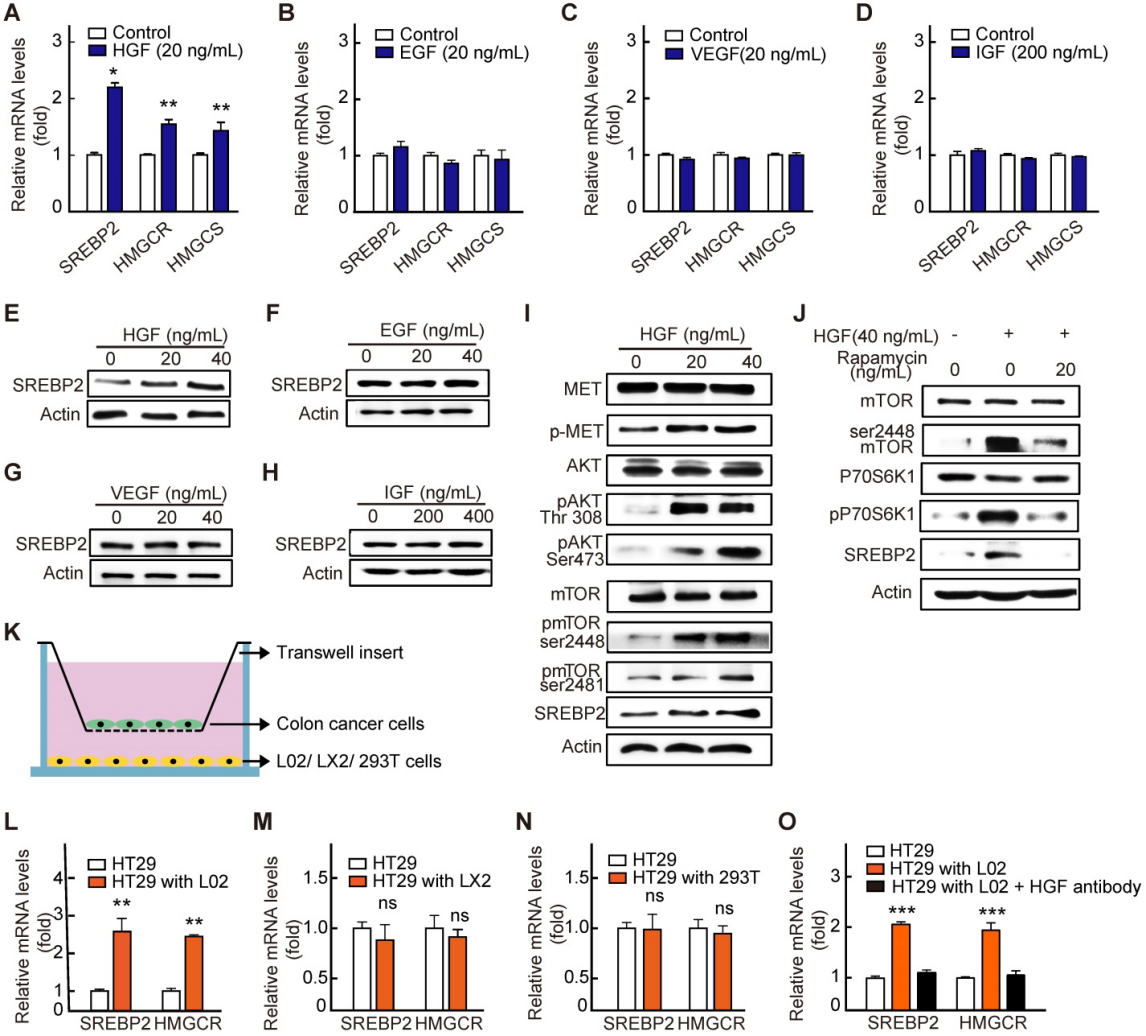
** HGF up-regulates SREBP2 expression in liver metastases by activating c-Met/mTOR pathway. A-D,** RT-qPCR analysis of mRNA levels for genes involved in cholesterol biosynthesis pathway in HT29 treated with HGF (A), EGF (B), VEGF (C) and IGF (D). Representative results from at least three independent experiments are shown. Data are shown as mean ± SD of triplicate experiments. **E-H,** Immunoblot of SREBP2 protein in corresponding group in A-D. **I-J,** The effect of HGF treatment on the expression of SREBP2 and the phosphorylation of c-Met, AKT, and mTOR in HT29 cells without (I) or with Rapamycin (J). Representative results from at least three independent experiments are shown. **K-O,** A diagram showing the co-culture experiment system (K). RT-qPCR analysis of mRNA levels for *SREBP2* and *HMGCR* in HT29 cells co-cultured with hepatocyte cell line L02 (L), hepatic stellate cell lines LX2 (M), human embryonic Kidney cell line 293T (N) or L02 supplemented with HGF antibody (O). Representative results from two biological independent experiments are shown. Data are shown as mean ± SD of triplicate experiments. Significance was determined by student's *t*-test (A-D, I-N) or one-way ANOVA (O). *P < 0.05, **P < 0.01, ***P < 0.001. ns, not significant.

Next, to reveal the mechanism underlying the up-regulation of SREBP2 expression in liver metastases of CRC, we first performed a screening for liver-rich growth factors including HGF, VEGF, IGF and EGF. The results showed that HGF, rather than other growth factors, significantly increased the SREBP2 expression in HT29 cells (Figure 5A-H). As reported previously, SREBP2 expression is controlled by mTORC1 signaling pathway 28, 29, and HGF can activate mTORC1 through c-Met/PI3K/AKT pathway 30. We then examined the activation of c-Met/PI3K/AKT/mTOR axis in HT29 cells under the treatment of HGF. As expected, with the treatment of HGF, the phosphorylation levels of c-Met, AKT and mTOR were all significantly increased (Figure 5I). Subsequently, rapamycin, the mTOR inhibitor, was used. Rapamycin treatment hindered the effect of HGF treatment on increasing phosphorylated mTOR and SREBP2 expression (Figure 5J), indicating that HGF upregulates SREBP2 expression through c-Met/PI3K/AKT/mTOR axis.

To further explore the source of HGF in liver environment, we conducted co-culture experiment of tumor cell and cells in liver microenvironment including the hepatic parenchymal cell line L02 cells and human hepatic stellate cell line LX2 cells (Figure 5K). We found that the expressions of SREBP2 and its target gene HMGCR were significantly increased in HT29 cells only when HT29 cells were co-cultured with the L02 cells (Figure 5L). While no significant changes were observed when HT29 cells were co-cultured with LX2 cells (Figure 5M) or with non-hepatic cell 293T (Figure 5N). Furthermore, the HGF antibody treatment hindered the increased expression of SREBP2 and HMGCR genes in HT29 cells when co-cultured with L02 cells (Figure 5O). Taken together, these results support the notion that HGF from liver microenvironment induces cholesterol biosynthesis pathway by activating c-Met/PI3K/AKT/mTOR axis in liver-metastatic CRC cells.

### Targeting cholesterol biosynthesis pathway suppresses liver metastasis of CRC

**Figure 6:**
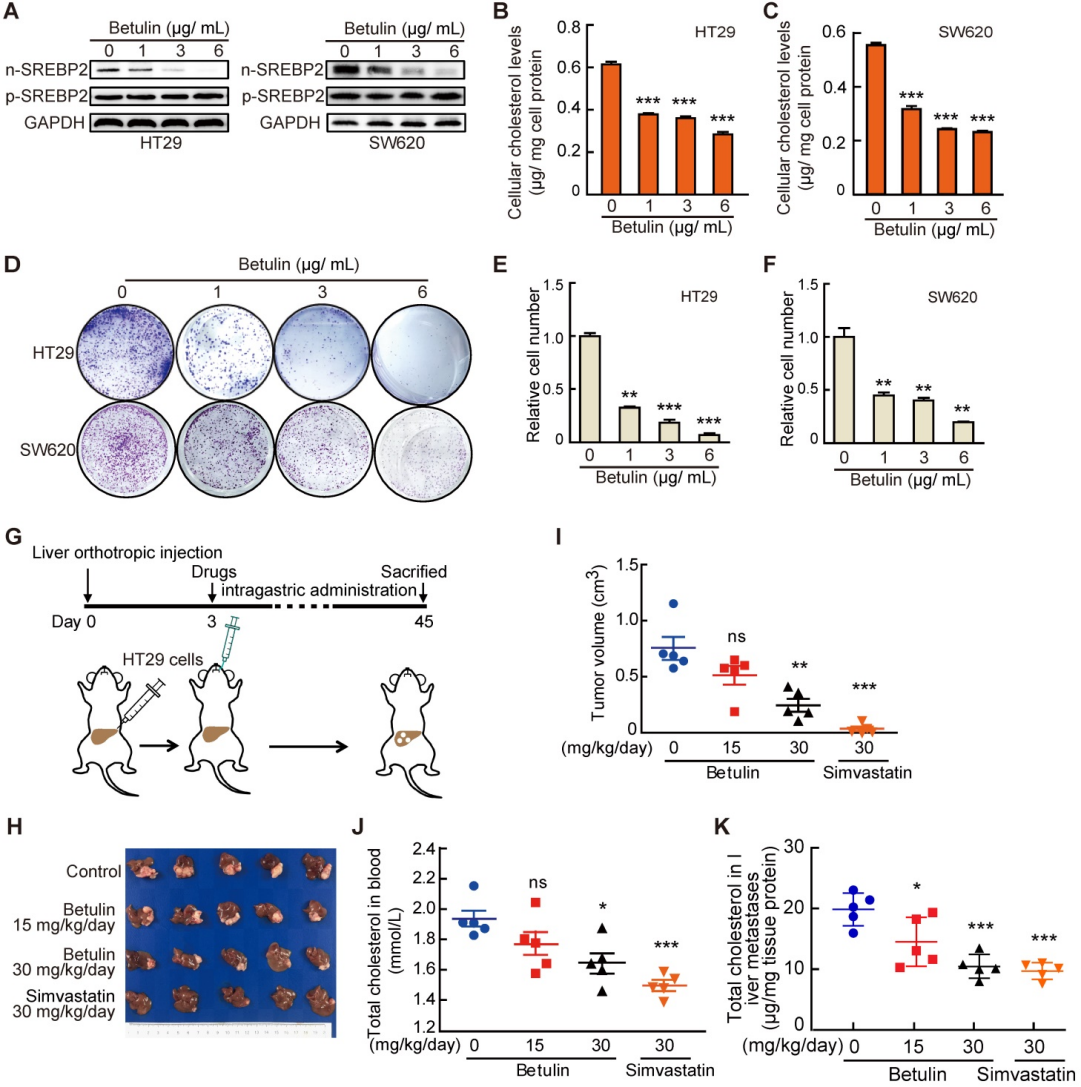
** Targeting cholesterol biosynthesis pathway suppresses CRC liver metastasis. A-C,** The effect of Betulin on expression of nuclear form of SREBP2 (n-SREBP2) and precursor of SREBP2 (pre-SREBP2) (A), total cholesterol level (B, C) and colony formation (D-F) in HT29 and SW620 cells. Representative results from three biological independent experiments are shown. Data are shown as mean ± SD of triplicate experiments (B-E). **G-K,** A schematic diagram showing the experiment design (G). HT29 cells were injected into liver of mice. Three days after injection, mice were administered with betulin or simvastatin as indicated. At the end of the experiment, liver metastases were taken photos (H) and were dissected for assessing tumor volume (I), and total cholesterol levels were determined in serums (J) and in liver metastases (K). Data are shown as mean ± SEM (n = 5, I-K). Significance was determined by one-way ANOVA (B-E, I-K). *P < 0.05, **P < 0.01, ***P < 0.001. ns, not significant.

Finally, to further explore the potential value of targeting SREBP2-dependent cholesterol biosynthesis pathway in treating liver metastasis of CRC, we used Betulin 31, a specific small-molecule inhibitor of SREBP, and Simvastatin, an inhibitor of HMGCR which is the rate-limiting enzyme in cholesterol biosynthesis, to intervene the SREBP2-dependent cholesterol biosynthesis pathway. Firstly, we examined the effects of betulin on the total cholesterol level and the growth of CRC cells *in vitro*. As expected, betulin treatment led to obvious decreases of nuclear form of SREBP2 (n-SREBP2) (Figure 6A) and cellular cholesterol content (Figure 6B-C) in both HT29 and SW620 cells. At the same time, betulin suppressed cell growth and colony formation of both HT29 and SW620 cells (Figure 6D-F). In liver orthotopic implantation model, betulin significantly inhibited the growth of liver metastases of CRC in a higher dose (Figure 6G-I). A more significant suppressive effect could be observed upon the administration of simvastatin (Figure. 6G-I). We further determined the total cholesterol levels in serum and liver metastases, and found that the administration of betulin (higher dose) or simvastatin resulted in significant decrease of total cholesterol level in mice serum (Figure 6J) and in liver metastases (Figure 6K), which confirmed the inhibitory effects of betulin or simvastatin on SREBP2-dependent cholesterol biosynthesis pathway. Similar results were also obtained by using another experimental CRC liver metastasis model by intrasplenic injection (Figure S6). Hence, targeting cholesterol biosynthesis pathway has potential therapeutic value for combating liver metastases of CRC.

## Discussion

Recently, Teng et al. have showed that liver metastatic CRC cells gain a liver-specific gene transcription program 20. In our study, we have shown that the activation of SREBP2-dependent cholesterol biosynthesis pathway was observed specifically in the liver metastases, but not in brain metastases and lymph node metastases, suggesting that metastatic CRC cells gain a liver-specific cholesterol metabolic characteristic. Several previous literatures have reported that SREBP2 expression is elevated and promotes cancer progression in several cancers including breast cancer, pancreatic cancer and prostate cancer 32-34. However, it has not been determined whether SREBP2 is elevated in metastases of these cancers. Interestingly, clinical studies showed that the serum total cholesterol level was higher in metastatic prostate cancer than that in non-metastatic prostate cancer 35, and that high serum HDL cholesterol level was associated with distant metastasis of gallbladder cancer 36. Chen et al. reported that targeting cholesterol transport by trapping cholesterol in lysosomes in circulating melanoma cells could inhibit melanoma metastasis 37. These studies suggested that cholesterol metabolic alteration do play roles in cancer metastasis. In addition to CRC, the liver is also the metastatic organ of other cancers including pancreatic cancer, gastric cancer, breast cancer, gallbladder adenocarcinoma, and squamous cell carcinoma of the esophagus 38, 39. So, it will be interesting to determine whether SREBP2-dependent cholesterol metabolic alteration also exists in liver metastases of these cancers.

Besides de novo biosynthesis, cells can also intake cholesterol by LDLR and SRB1 in liver 40. We did observe the elevated expression of *LDLR* and *SRB1* in liver metastases compared to that in primary tissues (Figure 1B). However, the expression of *LDLR* and *SRB1* in CRC liver metastases were much lower those in matched adjacent normal liver tissues (Figure S7), suggesting that de novo biosynthesis may be the main cholesterol source for liver metastases of CRC. Therefore, targeting these organ-specific metabolic pathways maybe a future direction for treating cancer metastasis. Statins, as inhibitors of HMGCR, have widely used as therapeutic drugs to reduce plasma cholesterol levels. Multiple studies have shown an inverse relationship between statin use and cancer risk, also indicating a direct association with cancer-related survival 41, 42. Our study provides evidence for the potential application of statins in treating the liver metastases of CRC patients with liver metastasis. However, it should be pointed out that the effect of Simvastatin in cancer prognosis is controversial. On one hand, a cohort study of Danish breast cancer patients showed that simvastatin use had a protective effect on breast cancer recurrence 43, and a meta-analysis indicated that usage of statins led to reduction of HCC risk 44. On the other hand, a meta-analysis included 19 studies indicated that statin use did not lower the risk of ovarian cancer or endometrial cancer 45.

Bu et al. showed that CRC cells undergo aldolase B-mediated fructose metabolic alternations in liver environment 19. Our findings showed that CRC cells also undergo cholesterol metabolic alternations when metastasize to liver, which is required for the growth of liver metastases. Intriguingly, we also observed a few differentiated genes involved in other metabolic pathways, such as *MGAT1* and *LIPE* involved in triglyceride metabolism (Figure S1). The roles of these metabolic alternations in liver metastasis of CRC needed further study.

Increasing evidences have shown that metastatic tumors frequently exhibit altered metabolic pattern compared to their primary tumor counterparts 12. However, whether these metabolic alterations result from selection of specific subset of primary tumor cells or from the adaptations of tumor cells to microenvironment of metastatic organ remains an open question. In our study, we showed that HGF from liver environment is responsible for the activation of cholesterol biosynthesis pathway in liver metastases of CRC, which supports the model that tumor cells undergo dynamical metabolic adaptation to local conditions of metastatic organ.

In conclusion, our study demonstrates that a CRC liver metastasis-specific cholesterol metabolic pathway that involves the activation of SREBP2-dependent cholesterol biosynthesis is established for colonization of metastatic CRC cells in the liver. Inhibiting this cholesterol biosynthesis pathway suppresses CRC liver metastasis. Mechanically, HGF from liver environment upregulates SREBP2 in CRC cells by activating c-Met/PI3K/AKT/mTOR axis. Collectively, targeting cholesterol biosynthesis pathway may be a promising therapy for liver metastasis of CRC.

## Supplementary Material

Supplementary figures and tables.
