# Supplementary material for: Organ-specific cholesterol metabolic aberration fuels liver metastasis of colorectal cancer
Source: Theranostics. 2021 Apr 27;11(13):6560–72. doi: 10.7150/thno.55609 (PMC8120208; doi:10.7150/thno.55609)
Supplement: Supplementary file 1 — Supplementary figures and tables. [file thnov11p6560s1.pdf]

Supplementary Data

Table S1. Clinicopathological information of CRC patients with liver metastasis

Case	Sex	Age (yrs)	Location	Number of LM	Histological grade	TNM stage
1	M	57	Sigmoid colon	1	II	pT3N1aM1a IVA
2	F	44	Ascending colon	1	II	pT3N2aM1a IVA
3	M	62	Rectum	1	II	pT3N1aM1a IVA
4	M	52	Rectum	1	III	pT3N1aM1a IVA
5	F	43	Decending colon; Sigmoid colon	3	III	pT3N1aM1a IVA
6	F	53	Ascending colon	2	II	pT3N2aM1a IVA
7	M	64	Ascending colon	1	II	pT3N1aM1a IVA
8	F	27	Rectum	1	II	pT3N1aM1a IVA
9	M	46	Decending colon	1	II	pT3N1aM1a IVA
10	F	71	Ascending colon	1	II	pT3N2aM1a IVA
11	M	49	Ascending colon	1	III	pT3N1aM1a IVA
12	M	79	sigmoid colon	1	II	pT3N2aM1a IVA
13	F	56	Ascending colon	1	III	pT3N2aM1a IVA
14	F	35	Ascending colon	1	II	pT3N1aM1a IVA
15	M	58	sigmoid colon	7	II	pT3N2aM1a IVA
16	M	51	sigmoid colon	1	II	pT3N2aM1a IVA
17	M	31	Rectum	3	II	pT3N2aM1a IVA

Abbreviation: LM, liver metastasis.

Table S2. Sequences of primer sets used in quantitative RT-PCR

Gene	Gene Bank Accession NO.	Sequence(5' to 3')
SREBP2	NM_004599	CTCACCTTCCTGTGCCTCTC
		AGGCATCATCCAGTCAAACC
HMGCR	NM_001130996	GTCATTCCAGCCAAGGTTGT
		CATGGCAGAGCCCACTAAAT
HMGCS	NM_001098272	GATGGACGGTATGCCCTGGTAT
		CTCCACCTGTAGGTCTAGCATG
SRB1	NM_001082959.2	GCTCGGAGAGCGACTACATC
		CCACATGATCTCACCCACAG
LDLR	NM_001010978	GCTTGTCTGTCACCTGCAAA
		AACTGCCGAGAGATGCACTT
ACAT1	NM_000019	GATGAAGGAAGGCTGGTGC
		GGAAGCTGGTGGCAGTGTAT
ACAT2	NM_005891	CATGCTGCTGCTCATCTTCT
		ACTGCGGAGACCAGGAACA
ABCA1	NM_080282	AACAGTTTGTGGCCCTTTTG
		AGTTCCAGGCTGGGGTACTT
ABCG1	NM_207174	ACGCAGTTCTGCATCCTCTT
		CGGAGTTGCTCAAGACCTTC
CYP7A1	NM_000780	CACCTTGAGGACGGTTCCTA
		CGATCCAAAGGGCATGTAGT
CYP27A1	NM_000784	AAGCGATACCTGGATGGTTG
		TGTTGGATGTCGTGTCCACT
β-ACTIN	NM_001101	GAGCTACGAGCTGCCTGACG
		CCTAGAAGCATTTGCGGTGG
ATGL	NM_020376	GGCTTCCTCGGCGTCTACTA
		TTTACCAGGTTGAAGGAGGGG
LIPE	NM_005357	TCAGTGTCTAGGTCAGACTGG
		AGGCTTCTGTTGGGTATTGGA
PLPP1	NM_003711	GGCAGGTTGTCCTTCTATTCAG
		CAGTGTGGGGCGTAAGAGT
MGAT1	NM_001114619	GTGCATAACCGGCCCGAATA
		ACGAGGACGTTGTCAATTCCC
AGPAT6	NM_178819	GGTATCCGCAAACTCTACATGAA
		CCACTTCGACGAATCTCTTTGA
MGLL	NM_001003794	ATGCCAGAGGAAAGTTCCCC
		CGTCTGCATTGACCAGGTG
PFK	NM_002626	GCATGGGTATCTACGTGGGG

		CTCTGCGATGTTTGAGCCTC
PGK1	NM_000291	GAACAAGGTTAAAGCCGAGCC
		GTGGCAGATTGACTCCTACCA
PKM	NM_182471	ATAACGCCTACATGGAAAAGTGT
		TAAGCCCATCATCCACGTAGA
G6PD	NM_000402	CGAGGCCGTCACCAAGAAC
		GTAGTGGTCGATGCGGTAGA
ENO2	NM_001975	AGCCTCTACGGGCATCTATGA
		TTCTCAGTCCCATCCAACTCC
HK1	NM_033498	GCTCTCCGATGAAACTCTCATAG
		GGACCTTACGAATGTTGGCAA
HK2	NM_000189	GAGCCACCACTCACCCTACT
		CCAGGCATTCGGCAATGTG
LDHA	NM_001165415	ATGGCAACTCTAAAGGATCAGC
		CCAACCCCAACAACTGTAATCT

Table S3. shRNA targeting sequence

Target gene	shRNA number	Sequence
SREBP2	shRNA#1	GCCCTCTATTGGATGATGCAA
	shRNA#2	GCAACAACAGACGGTAATGAT
	shRNA#3	GACCTGAAGATCGAGGACTTT

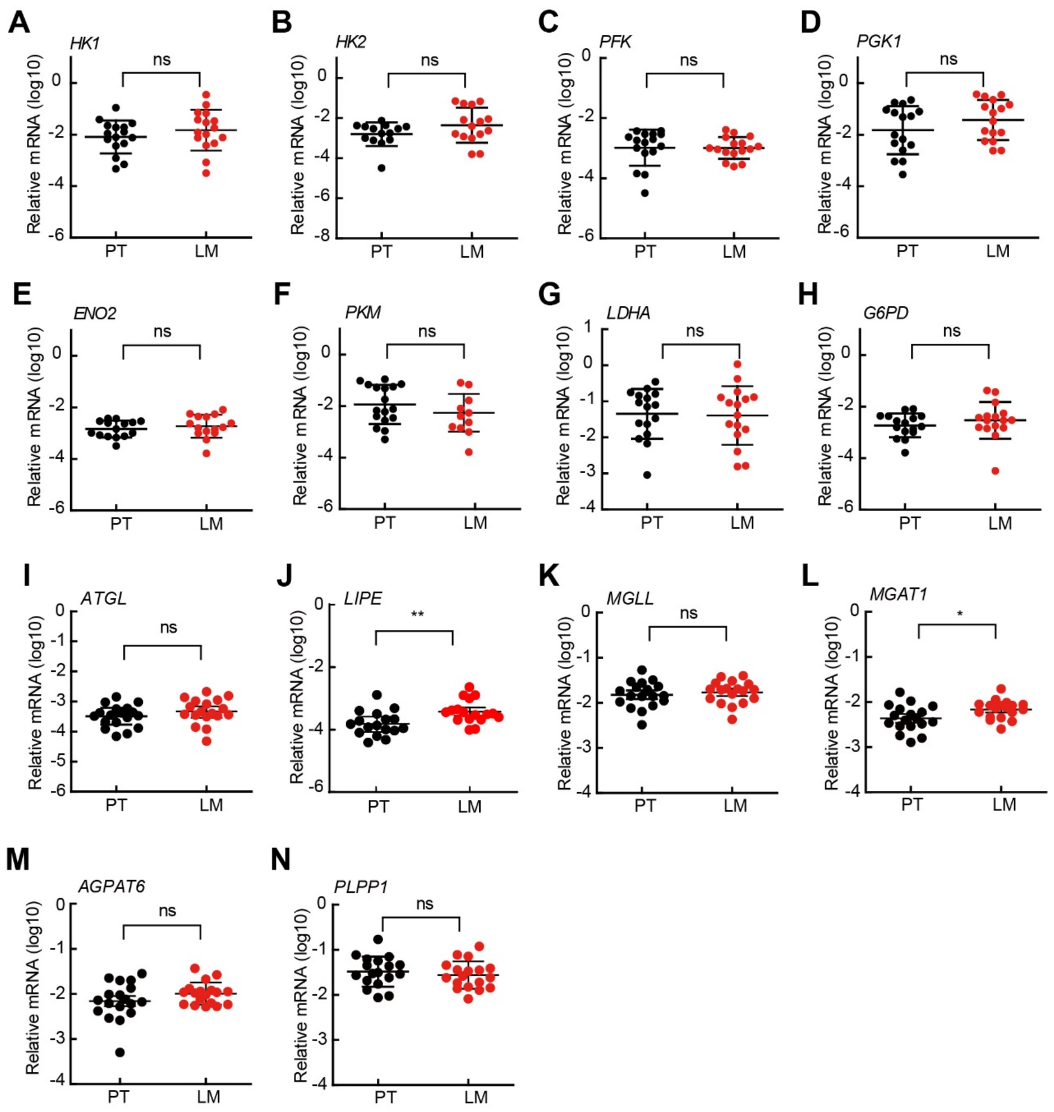

Figure S1. Expression of key genes involved in glycometabolism and triglyceride metabolism in liver metastasis of colorectal cancer

A-H, Quantitative RT–PCR analysis of mRNA levels for genes involved in glycometabolism and triglyceride metabolism in 17 paired samples from patients diagnosed with primary colorectal cancer (CRC) and liver metastasis. Primary tumor tissues (PT) and paired liver metastasis tissues (LM). Data are shown as mean ± SEM after log transformation (n = 17). Each dot represents the mean of relative mRNA level (log10) in triplicates for the indicated gene in each tissue sample.

I-N, Quantitative RT–PCR analysis of mRNA levels for triglyceride metabolism-related genes in 17 paired samples from patients diagnosed with primary colorectal

cancer and liver metastasis. Data are shown as mean ± SEM after log transformation (n = 17). Each dot represents the mean of relative mRNA levels (log10) in triplicates for the indicated gene in each tissue sample.

Significance was determined by a two-tailed paired *t*-test (**A-N**). *P < 0.05, **P < 0.01, ***P < 0.001, ns, not significant.

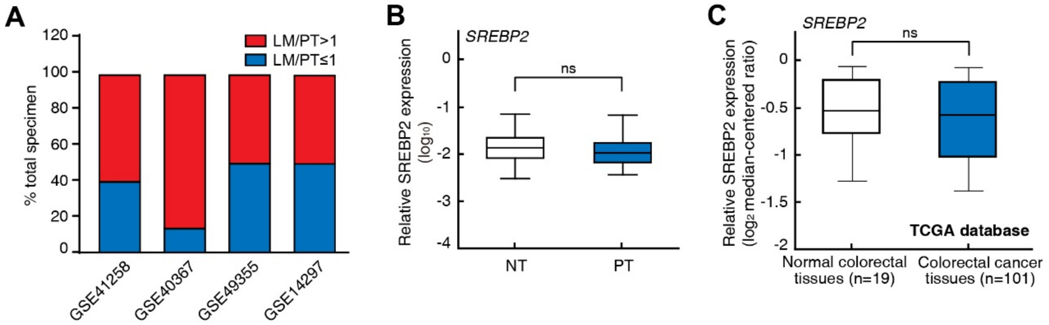

Figure S2. SREBP2 expression in CRC tissues

A, Relative SREBP2 expression in paired samples of PTs and LMs from 4 GEO datasets. The relative SREBP2 expressions in PTs and LMs from 4 GEO datasets are calculated by dividing the intensity of *SREBP2* to that of internal control gene *GAPDH*, and were then used to calculate the LM/PT ratio.

B, Relative SREBP2 mRNA levels in PTs and paired adjacent non-tumorous colorectal tissues (NTs). Data are shown as mean ± SEM after log transformation (n = 13). Triplicates conducted in each tissue sample.

C, Relative SREBP2 mRNA levels in PTs and NTs from TCGA database.

For (**B**) and (**C**), Significance was determined by a two-tailed paired *t*-test. $*P < 0.05$, $**P < 0.01$, $***P < 0.001$, ns, not significant.

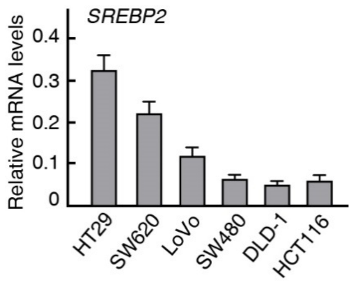

Figure S3. The mRNA levels of SREBP2 in different colon cancer cell lines.

RT-qPCR analysis of mRNA levels for SREBP2 involved in cholesterol biosynthesis pathway in 6 colon cancer cell lines, including HT29, SW620, LoVo, SW480, DLD-1 and HCT116. Representative results from at least three independent experiments are shown. Data are shown as mean ± SD of triplicate experiments.

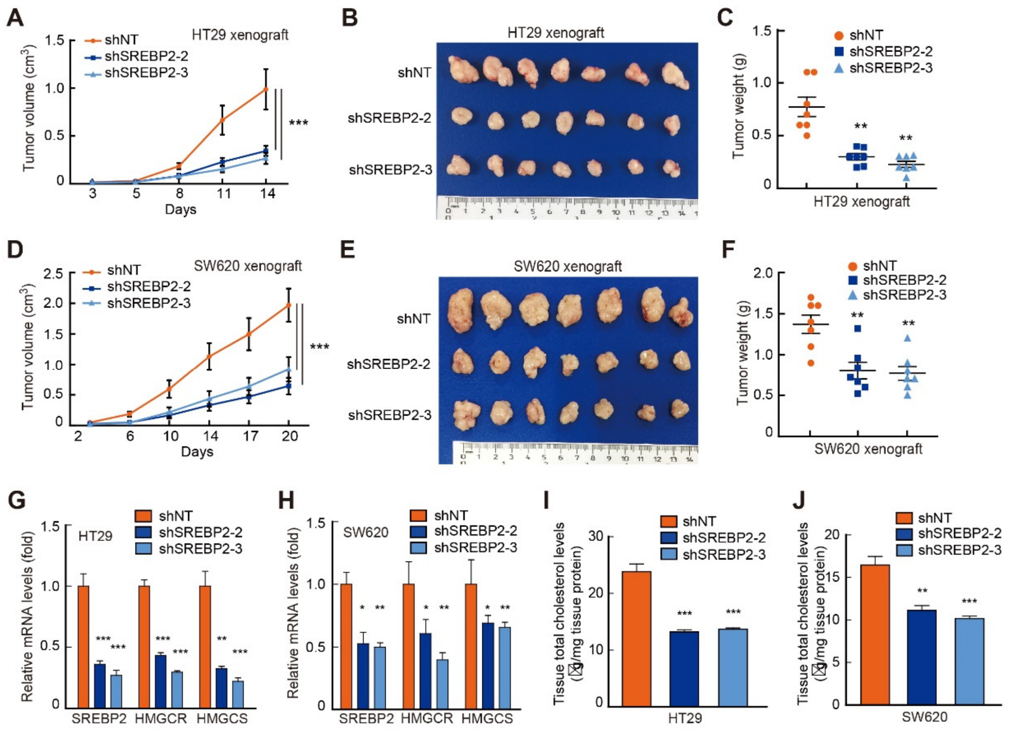

Figure S4. Knockdown of SREBP2 inhibits CRC tumor growth *in vivo*

A-F, Effects of SREBP2 knockdown on CRC xenograft tumor growth. Different HT29 or SW620 stable cell lines were used to establish xenograft tumors in nude mice. Tumor volumes were assessed on the indicated days (**A** and **D**) and tumors were dissected for photo (**B** and **E**) and weight (**C** and **F**). Data are shown as mean ± SEM. Significance was determined by two-way ANOVA (n = 7) (**A** and **D**) or one-way ANOVA (n = 7) (**C** and **F**).

G-J, Confirmation of *SREBP2* knockdown in HT29 or SW620 xenograft tumors. The expression of *SREBP2*, *HMGCR* and *HMGCS* (**G-H**) and total cholesterol content (**I** and **J**) were determined in HT29 and SW620 xenograft tumors. Representative results from at least three independent experiments are shown. Data are shown as mean ± SEM of triplicate experiments. Significance was determined by one-way ANOVA. **P < 0.01, ***P < 0.001.

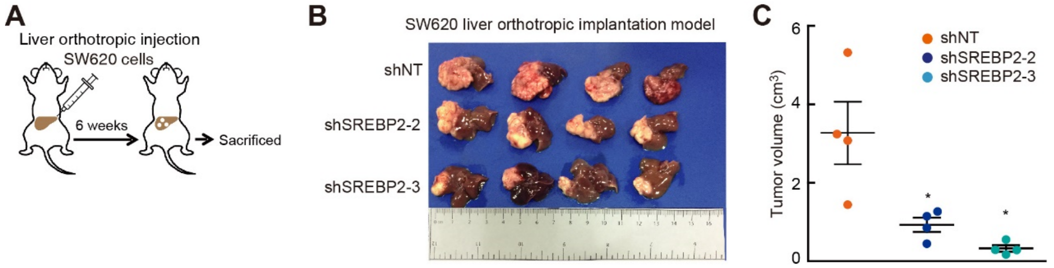

Figure S5. Knockdown of SREBP2 inhibits CRC liver metastasis *in vivo*

A-C, The effect of SREBP2 silencing on CRC liver metastasis in liver orthotropic injection model. Different SW620 stable cell lines were injected into liver of mice (**A**). At the end of the experiment, liver metastases were taken photos (**B**), and were dissected for assessing tumor volume (**C**). Data are shown as mean ± SEM (n = 4). Significance was determined by one-way ANOVA. *P < 0.05.

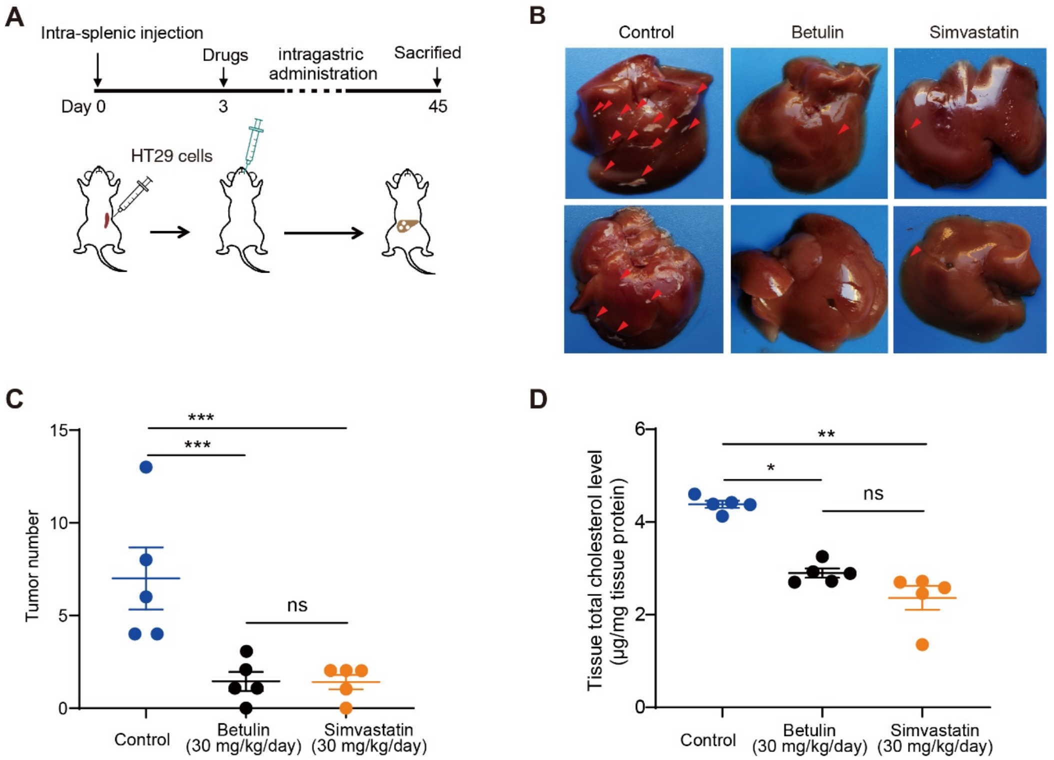

Figure S6. Cholesterol biosynthesis pathway is required for CRC liver metastasis.

The effect of betulin and simvastatin on CRC liver metastasis in intrasplenic injection model. Schematic diagram of intrasplenic injection mice model was presented in (**A**). Representative pictures of group Con/Betulin/Simvastatin, Red arrow indicates liver metastases (**B**) and metastatic lesion numbers were counted (**C**), and liver metastases tissues were dissected for assessing total cholesterol (**D**). Data are shown as mean ± SEM (n = 5). Significance was determined by one-way ANOVA. *P < 0.05, **P < 0.01, ***P < 0.001.

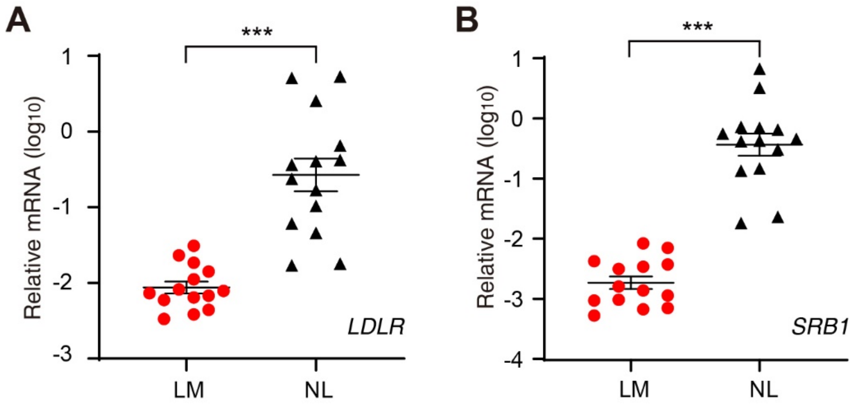

Figure S7. Evaluation of the cholesterol biosynthesis and influx pathway in CRC liver metastasis tissue (LM) and normal liver tissue (NL)

Quantitative RT–PCR analysis of mRNA levels for LDLR (**A**) and SR-B1 (**B**) in LM and paired NL**.** Data are shown as mean ± SEM after log transformation (n = 14). Each dot represents the mean of relative mRNA levels (log10) in triplicates for the indicated gene in each tissue sample.

Significance was determined by a two-tailed paired *t*-test. ***P < 0.001.
